# iMETHYL: an integrative database of human DNA methylation, gene expression, and genomic variation

**DOI:** 10.1038/hgv.2018.8

**Published:** 2018-03-29

**Authors:** Shohei Komaki, Yuh Shiwa, Ryohei Furukawa, Tsuyoshi Hachiya, Hideki Ohmomo, Ryo Otomo, Mamoru Satoh, Jiro Hitomi, Kenji Sobue, Makoto Sasaki, Atsushi Shimizu

**Affiliations:** 1 Division of Biomedical Information Analysis, Iwate Medical University, Shiwa, Iwate, Japan; 2 Division of Biobank and Data Management, Iwate Medical University, Shiwa, Iwate, Japan; 3 Laboratory of Bioinformatics, Department of Molecular Microbiology, Faculty of Life Sciences, Tokyo University of Agriculture, Setagaya, Tokyo, Japan; 4 Department of Anatomy, School of Medicine, Institute for Biomedical Sciences, Iwate Medical University, Shiwa, Iwate, Japan; 5 Iwate Tohoku Medical Megabank Organization, Iwate Medical University, Shiwa, Iwate, Japan; 6 Iwate Medical University, Morioka, Iwate, Japan; 7 Division of Ultrahigh Field MRI, Institute for Biomedical Sciences, Iwate Medical University, Shiwa, Iwate, Japan

## Abstract

We launched an integrative multi-omics database, iMETHYL (http://imethyl.iwate-megabank.org). iMETHYL provides whole-DNA methylation (~24 million autosomal CpG sites), whole-genome (~9 million single-nucleotide variants), and whole-transcriptome (>14 000 genes) data for CD4^+^ T-lymphocytes, monocytes, and neutrophils collected from approximately 100 subjects. These data were obtained from whole-genome bisulfite sequencing, whole-genome sequencing, and whole-transcriptome sequencing, making iMETHYL a comprehensive database.

DNA methylation (DNAm) has a critical role in regulating gene expression. Recent epigenome-wide association studies in humans have revealed that locus-specific DNAm signatures are associated with susceptibility to different environmental exposures, intermediate phenotypes, and diseases.^1,2^ Hence, locus-specific DNAm signatures are potential biomarkers in the era of precision medicine.^3^ We recently found that CpG sites with large interindividual DNAm variation are more likely to be potential biomarkers,^4^ suggesting that a database of interindividual DNAm variation would be useful to determine target regions for future epigenome-wide association studies.

Several studies have surveyed interindividual DNAm variation^5^ using peripheral blood, which contains many different cell types, but they did not investigate cell-type-specific signatures.^6^ Only a few studies have reported interindividual DNAm variation using purified cells, such as neutrophils^7^ and monocytes.^8,9^ Because differences in DNAm profiles among cell types are greater than those among individuals,^4^ profiling of DNAm variation using purified cells is essential to revealing interindividual DNAm variation within a cell type. In addition, the DNAm profiling methods frequently used in previous studies (e.g., array-based and targeted bisulfite sequencing) cover a limited number of human autosomal CpG sites (2–13%).^4^ Accordingly, whole-genome bisulfite sequencing, which provides the highest coverage (~90%) of human CpG sites among currently available methods, is desirable for compiling an interindividual DNAm variation database.^4^

Here we report the development and release of “iMETHYL” (http://imethyl.iwate-megabank.org), an integrative database (methylome, transcriptome, and genome) featuring interindividual DNAm variation. iMETHYL provides summarized open data calculated in our previous study, which characterized interindividual DNAm variation in two principal blood cell types, CD4^+^ T-lymphocytes (CD4T) and monocytes, which were collected from a cohort of healthy subjects (102 CD4T subjects and 102 monocyte subjects; Table 1) by whole-genome bisulfite sequencing.^4^ In addition to DNAm analysis, we performed whole-genome sequencing and whole-transcriptome sequencing to comprehensively profile genomic variation and gene expression, respectively. Briefly, sequence reads were aligned to the human reference genome GRCh37/hg19 using BWA-MEM (ver. 0.7.5a-r405), and single-nucleotide variant (SNV) calling was conducted using the Genome Analysis Toolkit (GATK version 2.5-2). Gene annotation was performed using GENCODE release 19.^10^ Details regarding the methods of quality-control filtering, DNAm profiling, gene expression profiling, and variant calling were described by Hachiya *et al.*^4^ In addition to CD4T and monocytes, we isolated neutrophils from 94 subjects and performed whole-genome bisulfite sequencing, whole-genome sequencing, and whole-transcriptome sequencing (Table 1). All subjects were recruited as part of the Tohoku Medical Megabank Project, and they provided written informed consent to participate in our study. All subjects belonged to a single large cluster on a PCA plot that consisted of Japanese subjects of the 1000 Genomes Project and the Tohoku Medical Megabank Project (Supplementary Figure 1). The study was approved by the Ethics Committee of Iwate Medical University (HG H5-558 19). iMETHYL was implemented on a UNIX server with CentOS, Apache HTTP Server, and JBrowse 1.12.1.^11^

Based on the DNAm profiles, we estimated the average DNAm levels and variation for ~24 million autosomal CpG sites. iMETHYL provides information on interindividual DNAm variation that was calculated by two methods, i.e., standard deviation (SD) and reference interval (RI), which is defined as the difference between the 95th and 5th percentiles of the DNAm level among individuals.^4^ In addition, iMETHYL includes the average and SD of gene expression levels for >14, 000 genes and allele frequencies for ~9 million autosomal SNVs (Table 1). Statistics regarding age, sex, and database profiles used in iMETHYL are presented in Table 1. Furthermore, genomic annotation tracks, such as gene models, repetitive elements, CpG islands, and microarray probes, are available in the iMETHYL browser (Table 2).

iMETHYL was developed to provide an informative, easy-to-use resource that enables investigators to explore DNAm levels and the variability of potential biomarkers identified by epigenome-wide association studies or candidate gene approach studies. From the iMETHYL browser, regions of interest can be specified using gene symbols (GENCODE release 19), dbSNP ID, DNA methylation array probe ID, and genomic positions. The genome browser provides graphical views of genomic annotations and the average methylation level and variability (SD and RI) of each CpG site in each of the three human cell types (Figure 1a). In addition, tracks for the average expression level and SD of each gene for each cell type and allele frequencies of each SNV within 102 (CD4T), 102 (monocytes), and 94 (neutrophils) subjects are provided.

In the example shown in Figure 1a, the iMETHYL genome browser showed different tracks in the region flanking cg05575921, which is a DNAm biomarker for tobacco smoking^12,13^ located in the aryl-hydrocarbon receptor repressor (*AHRR*) gene. This DNAm biomarker is markedly demethylated in current smokers.^12,13^ Using iMETHYL, the average methylation level and variability of each CpG site in the three cell types (CD4T, monocytes, and neutrophils) are shown, and by selecting the bar in the CpG tracks, histograms of DNAm levels at this CpG site for each cell type appear in pop-up windows (Figure 1b–d). iMETHYL is also useful for investigating cell-type-specific DNAm variability. In the CpG site shown in Figure 1, the DNAm levels in CD4T were hypermethylated with a narrow distribution (Figure 1b), whereas broader distributions of DNAm levels were found in monocytes and neutrophils (Figure 1c and d).

Furthermore, investigators can use the browser to explore variability in gene expression and SNVs. For example, upon selecting the bar shown in the fragments per kilobase of exons per million mapped fragment tracks, a histogram of gene expression levels appears in the pop-up window. In addition, the average expression level and SD for each gene are shown. This information provides important clues into the functional relevance of known or putative DNAm biomarkers.

Data on the mean and variation of the DNAm level of each CpG site for each of the three cell types can be downloaded from the iMETHYL website so that users can find CpG sites of their own interest based on the DNAm level and variation or differences between cell types.

In summary, we constructed a public database, iMETHYL, that provides a reference for human DNAm variation. iMETHYL is the first database featuring interindividual DNAm variation based on high-coverage whole-genome bisulfite sequencing using purified CD4T, monocytes, and neutrophils. Because the data were obtained from apparently healthy subjects, the multi-omics genomic data provided by iMETHYL can be used as a reference control. Investigators can examine DNAm variation, gene expression, and SNVs at any specific region of the human genome, which can enable the identification of variable regions in the population to design assay probes for microarrays or targeted sequencing. iMETHYL provides multi-omics data for three different cell types to the scientific community. The iMETHYL browser will be a useful resource not only for researchers specializing in epigenomics but also for those interested in the interactive analysis of DNA methylation, gene expression, and genomic variation.

## Publisher's Note

Springer Nature remains neutral with regard to jurisdictional claims in published maps and institutional affiliations.

## Figures and Tables

**Figure 1:**
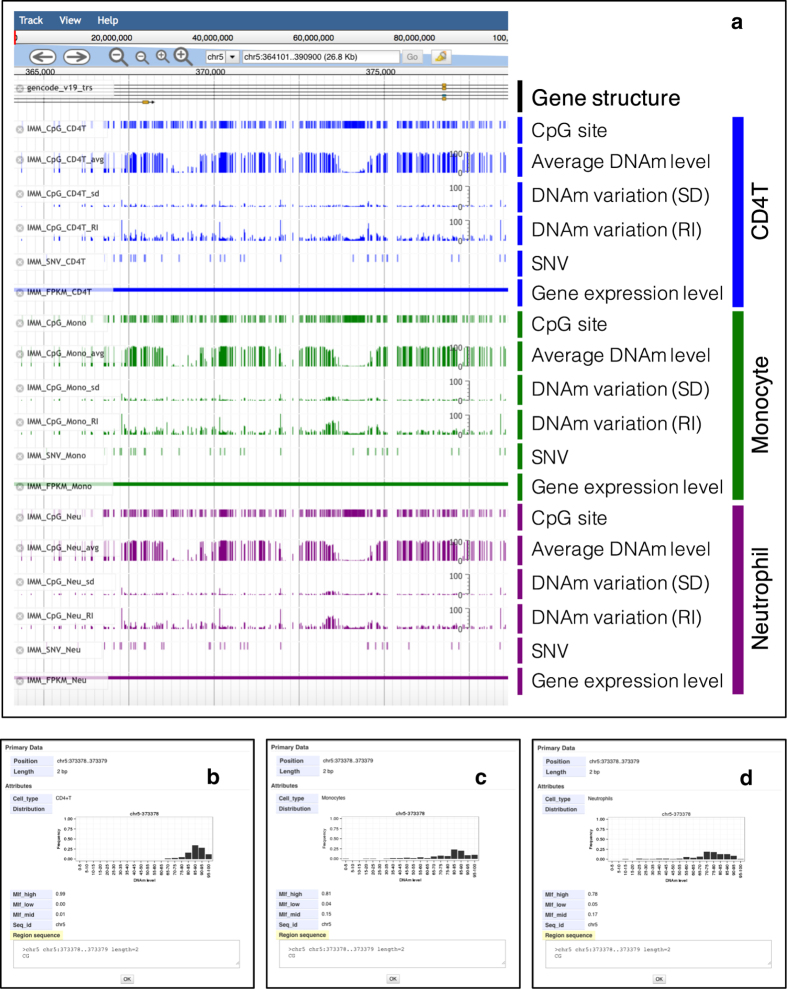
Graphical view of iMETHYL. (**a**) Three-layer omics data are provided as browser tracks. The browser displays several tracks, which are shown for the region surrounding the DNAm biomarker for tobacco smoking, cg05575921. Users can select tracks that provide information from external sources on gene structure, expression, and SNVs and cell-type-specific original tracks (e.g., CD4T, monocytes, and neutrophils) that show average DNAm levels and different measures of variation (SD and RI). (**b**–**d**) Detailed information on CpG tracks for CD4T, monocytes, and neutrophils. The frequencies of the three DNAm categories among individuals are shown as Mlf_high (≥ 67%), Mlf_mid (34–66%), and Mlf_low (≤ 33%). CD4T, CD4+ T-lymphocytes; DNAm, DNA methylation; Mlf_high, frequency of hypermethylated DNA; Mlf_high, frequency of hypermethylated DNA; Mlf_low, frequency of hypomethylated DNA; Mlf_mid, frequency of intermediate methylation DNA; RI, reference interval; SD, standard deviation; SNV, single-nucleotide variation.

**Table 1:** Demographic and profile statistics of iMETHYL

	Monocytes	CD4+ T cells	Neutrophils
*Demographic characteristics of subjects*			
* N*	102^a^	102^a^	94
Males, *N* (%)	48 (47.1)	49 (48.0)	48 (51.1)
Median age (range), years	62.5 (35–75)	62.0 (35–75)	58.0 (24–81)
			
*DNAm profiles*			
Sequencing depth^b^	31.1±1.8	31.0±1.6	54.7±1.6
No. of autosomal CpGs^c^	23 ,941,821	24,037,518	25,483,031
			
*Gene expression profiles*			
No. of sequencing reads^b^	33,917,157±3,153,528	35,175,996±1,275,575	47,040,140±6,289,540
No. of genes^d^	16,282	18,299	14,534
			
*SNV profiles*			
Sequencing depth^b^	27.2±1.0	27.2±1.0	53.3±13.2
No. of SNVs^e^	8,945,669	8,951,822	8,792,880

Abbreviations: DNAm, DNA methylation; SNV, single-nucleotide variant.

a Both cell types were obtained from the same 95 individuals out of a cohort of 102.

b Average±standard deviation.

c CpGs that were retained in ≥50% of subjects for each cell type.

d Genes that were expressed with a fragments per kilobase of exon per million mapped fragments ≥0.1 in ≥50% of subjects for each cell type.

e SNVs with a minor allele count >1.

**Table 2:** List of available tracks in iMETHYL

Track name	Description	Source
IMM_CpG_CD4T	Information for each CpG site of CD4T	Ref. ^4^
IMM_CpG_CD4T_avg	Average DNAm level of each CpG site of CD4T	Ref. ^4^
IMM_CpG_CD4T_sd	DNAm variations of each CpG site of CD4T measured by SD	Ref. ^4^
IMM_CpG_CD4T_RI	DNAm variations of each CpG site of CD4T measured by RI	Ref. ^4^
IMM_CpG_Mono	Information for each CpG site of monocytes	Ref. ^4^
IMM_CpG_Mono_avg	Average DNAm level of each CpG site of monocytes	Ref. ^4^
IMM_CpG_Mono_sd	DNAm variations of each CpG site of monocytes measured by SD	Ref. ^4^
IMM_CpG_Mono_RI	DNAm variations of each CpG site of monocytes measured by RI	Ref. ^4^
IMM_CpG_Neu	Information for each CpG site of neutrophils	This study
IMM_CpG_Neu_avg	Average DNAm level of each CpG site of neutrophils	This study
IMM_CpG_Neu_sd	DNAm variations of each CpG site of neutrophils measured by SD	This study
IMM_CpG_Neu_RI	DNAm variations of each CpG site of neutrophils measured by RI	This study
IMM_FPKM_CD4T	FPKM values of each transcript of CD4T	Ref. ^4^
IMM_FPKM_Mono	FPKM values of each transcript of monocytes	Ref. ^4^
IMM_FPKM_Neu	FPKM values of each transcript of neutrophils	This study
IMM_SNV_CD4T	Information for each SNV of CD4T	Ref. ^4^
IMM_SNV_Mono	Information for each SNV of monocytes	Ref. ^4^
IMM_SNV_Neu	Information for each SNV of neutrophils	This study
Reference sequence	Human genome hg19/GRCh37 sequence	UCSC genome browser
RepeatMasker	Repetitive elements	UCSC genome browser
CpGIslandsExt	CpG island locations	UCSC genome browser
HM450	Probe information for Illumina Infinium HumanMethylation450	UCSC genome browser
gencode_v19	Information of genes obtained from GENCODE version 19	GENCODE
gencode_v19_trs	Information of transcripts obtained from GENCODE version 19	GENCODE

Abbreviations: CD4T, CD4+ T-lymphocyte; DNAm, DNA methylation; FPKM, fragments per kilobase of exon per million fragments mapped; RI, reference interval; SD, standard deviation; SNV, single-nucleotide variant.

## References

[bib1] Rakyan VK, Down TA, Balding DJ, Beck S. Epigenome-wide association studies for common human diseases. Nat Rev Genet 2011; 12: 529–541. 21747404 10.1038/nrg3000 PMC3508712

[bib2] Mill J, Heijmans BT. From promises to practical strategies in epigenetic epidemiology. Nat Rev Genet 2013; 14: 585–594. 23817309 10.1038/nrg3405

[bib3] Andersen AM, Dogan MV, Beach SR, Philibert RA. Current and future prospects for epigenetic biomarkers of substance use disorders. Genes (Basel) 2015; 6: 991–1022. 26473933 10.3390/genes6040991 PMC4690026

[bib4] Hachiya T. et al. Genome-wide identification of inter-individually variable DNA methylation sites improves the efficacy of epigenetic association studies. NPJ Genome Med 2017; 2: 11. 10.1038/s41525-017-0016-5 PMC5677974 29263827

[bib5] Taudt A, Colomé-Tatché M, Johannes F. Genetic sources of population epigenomic variation. Nat Rev Genet 2016; 17: 319–332. 27156976 10.1038/nrg.2016.45

[bib6] Reinius LE. et al. Differential DNA methylation in purified human blood cells: implications for cell lineage and studies on disease susceptibility. PLoS ONE 2012; 7: e41361. 22848472 10.1371/journal.pone.0041361 PMC3405143

[bib7] Chatterjee A. et al. Genome-wide DNA methylation map of human neutrophils reveals widespread inter-individual epigenetic variation. Sci Rep 2015; 5: 17328. 26612583 10.1038/srep17328 PMC4661471

[bib8] Shen H, Qiu C, Li J, Tian Q, Deng HW. Characterization of the DNA methylome and its interindividual variation in human peripheral blood monocytes. Epigenomics 2013; 5: 255–269. 23750642 10.2217/epi.13.18 PMC3874233

[bib9] Furukawa R. et al. Intraindividual dynamics of transcriptome and genome-wide stability of DNA methylation. Sci Rep 2016; 6: 26424. 27192970 10.1038/srep26424 PMC4872231

[bib10] Harrow J. et al. GENCODE: the reference human genome annotation for The ENCODE Project. Genome Res 2012; 22: 1760–1774. 22955987 10.1101/gr.135350.111 PMC3431492

[bib11] Buels. R et al. JBrowse: a dynamic web platform for genome visualization and analysis. Genome Biol 2016; 17: 66. 27072794 10.1186/s13059-016-0924-1 PMC4830012

[bib12] Tsaprouni LG. et al. Cigarette smoking reduces DNA methylation levels at multiple genomic loci but the effect is partially reversible upon cessation. Epigenetics 2014; 9: 1382–1396. 25424692 10.4161/15592294.2014.969637 PMC4623553

[bib13] Zeilinger S. et al. Tobacco smoking leads to extensive genome-wide changes in DNA methylation. PLoS ONE 2013; 8: e63812. 23691101 10.1371/journal.pone.0063812 PMC3656907

