# Supplementary material for: iMETHYL: an integrative database of human DNA methylation, gene expression, and genomic variation
Source: Hum Genome Var. 2018 Mar 29;5:18008–. doi: 10.1038/hgv.2018.8 (PMC5874393; doi:10.1038/hgv.2018.8)
Supplement: Supplementary Information [file hgv20188-s1.docx]

*Human Genome Variation*

**Supplementary Figure 1**

iMETHYL: an integrative database of human DNA methylation, gene expression, and genomic variation

Shohei Komaki, Yuh Shiwa, Ryohei Furukawa, Tsuyoshi Hachiya, Hideki Ohmomo, Ryo Otomo, Mamoru Satoh, Jiro Hitomi, Kenji Sobue, Makoto Sasaki, and Atsushi Shimizu

**Supplementary Figure 1.** A PCA (principal components analysis) plot of the genotype data of East Asians (n = 10,557). Genotypic data for CDX, CHB, CHS, JPT, and KHV were collected from the 1000 Genomes Project.^1^ Data for TMM10K were collected from the Tohoku Medical Megabank Project^2^ which consists of 9,856 residents of Miyagi and Iwate Prefectures, Japan.

1. The 1000 Genomes Project Consortium. A global reference for human genetic variation. *Nature* 2015; **526**: 68–74.
2. Hachiya T *et al*. Genome-wide meta-analysis in Japanese populations identifies novel variants at the *TMC6–TMC8* and *SIX3–SIX2* loci associated with HbA_1c_. *Sci Rep* 2017; **7**: 16147.
